# An exploration in mental health care: The launch of China's first psychiatric noninvasive brain‐computer interface (BCI) and neuromodulation clinic

**DOI:** 10.1002/gps3.70069

**Published:** 2026-10-15

**Authors:** Yanyan Wei, Huiru Cui, Junjuan Zhu, Chengfeng Chen, Zhenying Qian, Dan Zhang, Jijun Wang, Tianhong Zhang

**Affiliations:** ^1^ Shanghai Mental Health Center, Neuromodulation Center, Shanghai Engineering Research Center of Intelligent Psychological Evaluation and Intervention Shanghai Jiao Tong University School of Medicine Shanghai China

Mental health disorders constitute a pressing global public health challenge, with traditional pharmacotherapy often failing to address the unmet needs of patients—particularly those with refractory conditions or intolerance to medications.^1^ Current symptom‐based empirical treatment remains a blunt, one‐size‐fits‐all approach: diagnoses rely heavily on subjective symptom checklists and interventions are selected through trial and error, with little consideration of individual neurobiological variability. This crude paradigm stands in stark contrast to the leaps in modern brain science (e.g., functional neuroimaging for circuit mapping) and data analytics (e.g., artificial intelligence [AI]‐driven biomarker discovery), creating a critical disconnect between research advancements and clinical practice. The persistent gap between symptom‐based empirical treatment and the neurobiological roots of mental illness has long hampered therapeutic progress, driving the demand for technology‐driven precision approaches. Against this backdrop, the Shanghai Mental Health Center launched China's first dedicated psychiatric noninvasive brain‐computer interface (BCI)^2^ and neuromodulation clinic on 18 December 2025, providing an exploratory integrated clinical framework combining neuroimaging, data decoding and personalised neuromodulation for psychiatric intervention.

The clinic operates as an open‐access service, welcoming all individuals seeking nonpharmacological mental health interventions. All individuals with mental health‐related distress are eligible to book consultations via official appointment channels. During multidisciplinary assessment, clinicians will explicitly inform each patient that the noninvasive BCI neuromodulation scheme^3^ is customised to target their core subjective complaints and dominant symptomatic circuits. For instance, patients with schizophrenia may receive circuit‐specific stimulation targeting auditory hallucinations as their primary symptom; if the same patient also presents comorbid depressive episodes or severe obsessive‐compulsive symptoms, the decoding model will adjust stimulation targets sequentially or simultaneously to address all their main clinical demands. Its core strength lies in a multidisciplinary team comprising psychiatrists, neurologists, radiologists and specialised data‐decoding experts who collaborate seamlessly throughout the medical journey. These data decoding experts are engineers with rigorous backgrounds in computer science, artificial intelligence and biomedical engineering, affiliated with the Shanghai Engineering Research Center of Intelligent Psychological Evaluation and Intervention, bringing technical expertise that is rarely embedded directly within clinical psychiatric teams. The workflow begins with a comprehensive assessment of each patient's clinical manifestations, symptom history and personal treatment goals.

To unravel the neurobiological underpinnings of each patient's condition, a suite of noninvasive brain function assessments is conducted within two working days of the initial consultation. These include structural and resting‐state magnetic resonance imaging (MRI) to map brain anatomy and functional connectivity patterns, electroencephalography (EEG) evoked potentials to capture real‐time neural electrical activity and functional near‐infrared spectroscopy (fNIRS) to measure haemodynamic changes reflecting regional brain activation. The combined goal of these assessments is to translate subjective psychiatric symptoms into objective neurobiological markers, identifying specific brain regions or neural circuits implicated in the patient's condition—such as aberrant connectivity patterns linked to depression^4^ or hyperactivity in circuits associated with anxiety.^5^

Following data acquisition, the specialised decoding team employs advanced algorithms to analyse the multi‐modal brain function data. For patients presenting with depression, the focus is on quantifying functional connectivity between the dorsolateral prefrontal cortex (DLPFC) and subgenual anterior cingulate cortex (sgACC), key nodes in the emotional regulation network—this connectivity‐guided targeting framework originates from the landmark Stanford Neuromodulation Therapy (SNT) accelerated repetitive transcranial magnetic stimulation (rTMS) protocol, a validated circuit‐based precision stimulation paradigm for mood disorders.^6^ For those with anxiety‐related somatic symptoms, the algorithm pinpoints optimal targets within the right dorsomedial prefrontal cortex, a region critical for modulating somatic manifestations of anxiety.^7^ This decoding process transforms raw neural data into actionable insights, identifying personalised stimulation targets that would otherwise remain elusive with conventional diagnostic methods. The test results and identified neuromodulation targets are then communicated to the patient.

Precision neuromodulation is delivered via neuro‐navigated transcranial magnetic stimulation (TMS), a noninvasive technique that ensures accurate targeting of the identified brain regions. Prior to each session, individual structural MRI images are registered from the patient's native anatomical space to the real‐time coordinate space of the TMS navigation system and the coordinates of the optimal stimulation voxel are loaded into a TMS Navigator system.^8^ During treatment, patients can visualise the real‐time alignment between their brain structure and the stimulation target, enhancing transparency and engagement. The acute phase treatment consists of 30–50 sessions, administered as 5–6 daily sessions with at least 50‐min intervals to ensure neural recovery, over a 6–8‐day period. The accelerated intensive protocol is not a rigid, fixed regimen for all cases. Clinicians design humanised, flexible intervention schedules fully tailored to each patient's real‐life conditions, diagnosis, illness chronicity and comorbid status. Special arrangements are provided for specific populations, such as students: their full treatment course will be scheduled during school vacations, after full communication with patients and their families and by adjusting daily treatment density and total intervention cycles to balance therapeutic efficacy and daily life demands. The intensive accelerated regimen follows the Stanford protocol as the standard for maximum dose. For patients under 18 years of age, the total daily stimulation dose is halved, with 5 daily treatment sessions scheduled to ensure safety.^9^ All stimulation paradigms, including intermittent theta‐burst stimulation (iTBS), continuous theta‐burst stimulation (cTBS) and conventional rTMS, are implemented with unified standardised parameters. After the treatment course is completed, follow‐up brain function assessments are performed to objectively evaluate the effects of neuromodulation (figure 1).

**FIGURE 1:**
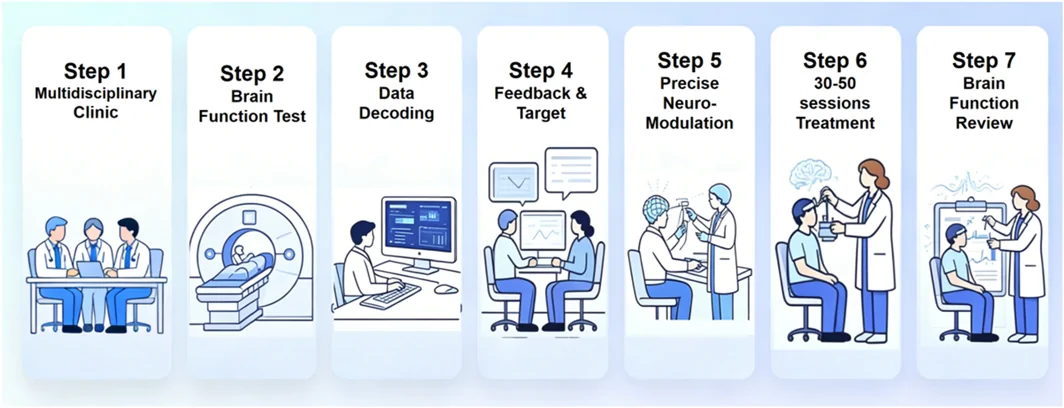
Workflow of noninvasive BCI and neuromodulation clinic. This figure illustrates the seven‐step clinical workflow for a noninvasive BCI and neuromodulation clinic. Step 1: Multidisciplinary joint clinic, including psychiatrists, neurologists, radiologists, and specialised data‐decoding experts, etc. Step 2: Multi‐modal brain function assessment, including structural MRI, resting‐state functional MRI, near‐infrared spectroscopy, electrophysiological recording and eye‐tracking. Step 3: Data decoding and verification by data analysts, integrating multi‐modal data with existing empirical targets. Step 4: Feedback on evaluation results and determination of personalised neuromodulation targets by the multidisciplinary team. Step 5: Precise neuromodulation targeting specific brain regions under neuro‐navigation guidance. Step 6: Completion of a full treatment course of approximately 30–50 sessions. Step 7: Follow‐up brain function evaluation to objectively assess the therapeutic effects of neuromodulation. BCI, brain‐computer interface; MRI, magnetic resonance imaging.

The clinic's launch also garnered unprecedented public attention: within one hour of the announcement, online views exceeded 10 000 and all initial appointment slots were fully booked within 10 minutes. To date, it has become the most difficult‐to‐book outpatient service at the Shanghai Mental Health Center—an unequivocal reflection of the widespread, unmet demand for safe, effective and personalised nonpharmacological mental health solutions in China.

This clinic earns its designation as a psychiatric BCI service by embodying the core principles of noninvasive BCI technology—an attribute that helps overcome critical barriers to psychiatric applications, where invasive procedures are often impractical or unacceptable due to patient safety concerns and stigma. All MRI, EEG and fNIRS assessments, as well as neuromodulation treatments, are standard routine clinical services for patients.

Conventional narrow BCI focuses on real‐time closed‐loop decoding of transient motor signals for rehabilitation. Psychiatric conditions are driven by stable pathological brain circuits that can last days to weeks, even years, justifying a broader open‐loop BCI framework here. We perform offline multi‐modal brain signal decoding to extract persistent disease biomarkers prior to personalised navigated TMS delivery—consistent with accepted open‐loop psychiatric BCI classifications in recent reviews.^3^, ^10^, ^11^ This pipeline differs fundamentally from basic image‐guided TMS, which lacks functional disease‐circuit decoding for individualised target localisation.

Specifically, the acquisition of brain signals through noninvasive MRI, EEG and fNIRS constitutes a safe, accessible ‘brain‐to‐machine’ communication pathway; the data‐driven analysis of these signals to identify personalised targets represents the critical ‘decoding’ step; and the delivery of neuro‐navigated neuromodulation based on this decoding achieves functional ‘brain‐computer interaction’ tailored to psychiatric needs. This noninvasive focus stands in deliberate contrast to traditional BCI, which has long been constrained by narrow definitions centred on invasive implants or direct brain‐machine control for motor rehabilitation^12^—applications that have little relevance to the complex, subjective nature of psychiatric disorders. By redefining BCI for psychiatry through a noninvasive lens, the clinic expands the technology's utility to a field where objective neural biomarkers and personalised interventions are urgently needed but have previously been lacking.

Three key barriers limit cross‐centre replication: scarce MRI at grassroots sites, site‐dependent decoding algorithms and public misconceptions of neuromodulation. We propose a tiered service network for scaled rollout. Top tertiary hospitals act as core hubs to perform MRI scans and AI‐personalised target decoding, then fabricate patient‐specific 3D positioning templates to fix stimulation coordinates. With these templates, patients receive follow‐up neuromodulation at nearby community centres without repeated MRI. Internet hospitals coordinate tiered care: top facilities unify algorithm standards, remotely supervise community treatment and deliver online public science, building an integrated multi‐level neuromodulation service network.

Beyond technical refinements, this clinic underscores a pivotal reality: BCI's transformative potential in psychiatry cannot be unlocked in academic silos. To fully realise its promise, the field must forge deeper, more collaborative intersections with industry partners—harnessing commercial research and development (R&D)‐driven innovations in portable, low‐cost neuroimaging tools, high‐speed, low‐latency data‐processing hardware and robust AI/machine‐learning decoding algorithms. This synergy between clinical academia and industry is not merely beneficial but essential: academia provides the clinical insights, patient‐centric goals and validation frameworks, whereas industry brings scalability, technological optimisation and resource mobilisation that accelerate the translation of experimental BCI prototypes into accessible, routine clinical tools. By fostering this bidirectional collaboration, psychiatry can move beyond incremental therapeutic improvements and towards leapfrog development—closing the long‐standing gap with other medical specialities that have already integrated technology‐industry partnerships to revolutionise precision care.

This trailblazing clinic represents a paradigm shift in psychiatric care, moving beyond symptom‐centric empirical treatment to neurobiology‐driven precision intervention. Its innovation lies in the seamless integration of noninvasive neuroimaging, data decoding and personalised neuromodulation into a scalable clinical service, rather than isolated technological application. This approach not only expands treatment options for patients but also advances the field towards a future where psychiatric care is as precise, objective and tailored as care in other branches of modern medicine. Notably, this work merely describes a clinical service model and lacks quantitative data on patient numbers, treatment efficacy, follow‐up results and adverse events. We cannot draw definitive conclusions about its safety or effectiveness. Large prospective cohorts with standardised outcomes, adverse event monitoring and long‐term follow‐up are needed to validate its clinical value.

## AUTHOR CONTRIBUTIONS

Tianhong Zhang, Yanyan Wei, Huiru Cui and Jijun Wang conceptualised the study, wrote the first draft of the manuscript and conducted the statistical analyses. Zhenying Qian, Chengfeng Chen, Junjuan Zhu and Dan Zhang reviewed and edited the manuscript. Tianhong Zhang and Jijun Wang designed the study and provided supervision during its implementation. All authors have approved the final manuscript.

## FUNDING

This work was supported by the Ministry of Science and Technology of China, National Key R&D Programme of China (2023YFC2506800), National Natural Science Foundation of China (82371505), Project of Shanghai Mental Health Center (2024‐QM‐02, 2022YJ09) and the Shanghai Municipal Health Commission Clinical Research Special Project (202440203).

## CONFLICT OF INTEREST STATEMENT

All authors report no biomedical financial interests. Dr Jijun Wang is an editorial board member of General Psychiatry and a co‐author of this article; Dr Wang was excluded from editorial decision‐making related to the acceptance of this article for publication in the journal.

## Data Availability

None.
